# Translation and psychometric evaluation of the German version of the IcanSDM measure – a cross-sectional study among healthcare professionals

**DOI:** 10.1186/s12913-021-06430-3

**Published:** 2021-06-02

**Authors:** Anja Lindig, Pola Hahlweg, Eva Christalle, Anik Giguere, Martin Härter, Olaf von dem Knesebeck, Isabelle Scholl

**Affiliations:** 1 Department of Medical Psychology, University Medical Center Hamburg-Eppendorf, Martinistraße 52, 20246 Hamburg, Germany; 2 Center of Health Care Research, University Medical Center Hamburg-Eppendorf, Martinistraße 52, 20246 Hamburg, Germany; 3 Laval University Research Centre on Community-Based Primary Health Care (CERSSPL-UL), Québec City, QC Canada; 4 Department of Medical Sociology, University Medical Center Hamburg-Eppendorf, Martinistraße 52, 20246 Hamburg, Germany

**Keywords:** Measuring attitudes regarding shared decision-making, Perspective of healthcare professionals, Psychometrics, Implementation, Shared-decision making, Measurement

## Abstract

**Background:**

Shared decision-making (SDM) between patients and healthcare professionals (HCPs) is a key component of patient-centred care. To implement SDM in clinical practice and to evaluate its effects, it is helpful to know about HCPs’ perception of SDM barriers. The measure IcanSDM was developed in Canada and assesses the perception of SDM barriers. To our knowledge, no equivalent measure exists in German. Therefore, the aim of this study was to translate and adapt the IcanSDM measure to be used by a German speaking population and evaluate its psychometric properties.

**Methods:**

This is a cross-sectional psychometric study based on a secondary analysis of baseline data from a SDM implementation study. The original 8-item IcanSDM was translated into German using a team translation protocol. We assessed comprehensibility via cognitive interviews with *n* = 11 HCPs. Based on results of cognitive interviews, the translated IcanSDM version was revised. Two hundred forty-two HCPs filled out the measure. Psychometric analysis included acceptance (completion rate), item characteristics (response distribution, skewness, item difficulties, corrected item-total correlations, inter-item correlations), factorial structure (confirmatory factor analysis (CFA), model fit), and internal consistency (Cronbach’s α).

**Results:**

We translated and adapted the German IcanSDM successfully except for item 8, which had to be revised after the cognitive interviews. Completion rate was 98%. Skewness of the items ranged between −.797 and 1.25, item difficulties ranged between 21.63 and 70.85, corrected item-total-correlations ranged between .200 and .475, inter-item correlations ranged between .005 and .412. Different models based on CFA results did not provide a valid factorial structure. Cronbach’s α ranged between .563 and .651 for different factor models.

**Conclusion:**

We provide the first German measure for assessing perception of SDM barriers by HCPs. The German IcanSDM is a brief measure with good acceptance. However, we found unsatisfying psychometric properties, which were comparable to results of the original scale. In a next step, the IcanSDM should be further developed and modified and predictive validity should be evaluated.

**Supplementary Information:**

The online version contains supplementary material available at 10.1186/s12913-021-06430-3.

## Background

Providing patient-centered care means to respect patients preferences, needs and values and reveals the importance of patients values for clinical decisions [1]. The integration of patient preferences into health care decisions can be realized by shared decision-making (SDM) [2–5]. SDM involves information exchange between healthcare professionals (HCPs) and their patients [6, 7]. HCPs and patients discuss different treatment options based on the best available evidence about the likely benefits and harms. Patients are supported to build informed preferences. Thereby, HCPs and patients are equally and actively involved in the decision-making process and jointly responsible for the decision [7–9]. SDM is especially important in situations in which different and often complex treatment options have a high impact on quality of life (so-called preference-sensitive decisions) [10]. Most patients prefer SDM [11] and benefit from SDM by having better knowledge about their disease and treatment options, less insecurity and decisional conflict and better risk perception [12, 13].

In many countries, the implementation of SDM is supported by public policies [14] and research [15–18]. Nevertheless, SDM is not widely and routinely implemented in the German healthcare system or healthcare systems of other countries yet [3, 19, 20]. A range of barriers that hinder SDM uptake have been reported [21, 22]. Barriers to SDM exist on the health system level (e.g. payment models), organizational level (e.g. lack of resources, lack of leadership support) and the individual level of HCPs and patients (e.g. resistance to change, negative attitudes towards SDM, perception of characteristics of specific clinical situations or patients) [23–26].

Predicting and explaining HCPs’ behaviour in the context of SDM uptake can help to develop successful interventions which overcome these barriers. The widely-tested Theory of Planned Behaviour (TPB) [27–29] can be helpful to predict and explain HCPs behaviour. According to the TPB, a central factor for behaviour is individuals’ intention to perform a behaviour. Behavioural intentions are determined by individuals’ attitudes towards the behaviour, subjective norms, and perceived behavioural control [29]. There is a substantial correlation between intention and behaviour [30]. If individuals’ intention to perform a specific behaviour is high, they are more willing to try and exert more effort. Additionally, non-motivational factors like availability of opportunities and resources can influence actual behavioural control and therefore the intention to perform a specific behaviour. According to Fishbein and Ajzen, adoption of a behaviour, like performing SDM, is influenced by knowledge, behavioural intention, and attitudes towards the behaviour [28]. Congruently, there are several studies conducting SDM trainings for HCPs which have shown to be effective in improving HCPs ability of, attitudes towards and confidence in SDM uptake [31–35]. To assess these outcomes, valid and reliable instruments are necessary. There is already a range of measures, which assess the SDM process (i.e. the communicative process between HCP and patient, through which a medical decision is achieved) and SDM outcomes (i.e. satisfaction with decision, decisional regret) [36]. Additionally, a few measures assess other aspects like barriers for SDM uptake [35]. To investigate, why individuals differ in SDM uptake, it is of interest to evaluate determinants of SDM uptake. One of those determinants is the perception of barriers on different levels (e.g. attitude, perceived behavioural control, social norms). HCPs’ perception of SDM barriers might indicate HCPs’ intention to perform SDM and can be an indicator for differences in SDM performance between HCPs.

Up to now, there is only one measure addressing HCPs’ perception of SDM barriers. This new measure IcanSDM was recently developed in Canada for a French-speaking population [35] and translated into English for publication. It measures the perception of barriers for the uptake of SDM and has been psychometrically evaluated by the Canadian study group, revealing a Cronbach’s α of above 0.63 and a trend to show sensitivity to change [35]. Although the IcanSDM is still at the beginning of its development and evaluation, it has the potential to be used to assess HCPs’ perception of SDM barriers in different healthcare systems. So far, the IcanSDM was not translated to other languages or further validated. In Germany, no comparable measure exists up to now. To be used in German SDM implementation studies, the original IcanSDM has to go through cross-cultural adaptation to reduce the risk of bias due to translation into a different language and assessments in a different culture and setting and at a different time [37]. To do so, items must not only be translated linguistically but have to be adapted culturally [38, 39]. Differences in the cultural context can lead to different validity. Thus, analysis of validity and reliability are necessary to ensure cross-cultural comparability of the measures [38].

Aim of this study was to translate and adapt the IcanSDM measure to be used by a German speaking population and evaluate its psychometric properties.

## Methods

### Study design

For the psychometric evaluation of the IcanSDM we conducted a secondary analysis of cross-sectional data. Cross-sectional data were taken from an implementation study to foster SDM uptake in a German healthcare setting [22]. In this implementation study, we used a stepped wedge design, a variant of a cluster randomized trial [40, 41]. Thereby, a multi-component SDM implementation program was implemented in three departments of a Comprehensive Cancer Center in Hamburg, Germany. Each department represented a cluster and all physicians and nurses within these clusters were included in the study. Each cluster was first observed under control conditions before it received the intervention in a randomized sequence. It was then observed under intervention condition until the end of the study [40, 41]. We performed an outcome evaluation at four measurement points as well as a process evaluation throughout all study phases [22]. The psychometric evaluation of the IcanSDM is a secondary analysis of baseline data (control condition) of this SDM implementation study. A waiver of consent for HCPs was obtained from the Ethics Committee, as proposed by current statements of ethical design of implementation research. Study participation was voluntary and HCPs were able to decline participation in the study by not filling out the survey.

To report the results of this study, we used the Authors’ Guidelines for Reporting Scale Development and Validation Results by Cabrera-Nguyen as well as the STROBE Statement for cross-sectional studies. For assessment of comprehensibility as part of content validity, we used the COSMIN criteria (Consensus-based standards for the selection of health measurement instruments).

We previously published the translation and psychometric evaluation of a second measure, the Organizational Readiness for Implementing Chance (ORIC), which was assessed together with the IcanSDM within the same survey [42]. The translation and adaptation procedure as well as the psychometric evaluation of the ORIC and the IcanSDM was done in parallel using very similar methods. For example, both measures were tested within the same cognitive interview sessions. Thus, there might be an overlap in the methods for both measures, ORIC and IcanSDM.

For an overview on all steps of this study, see Table 1.

**Table 1:** Overview of the process of this study

1	Translation according to TRAPD protocol	Translation: Two independent translations were produced.
		Review: A third bilingual reviewer compared the two translations and decided for one of the translations or suggested a third translation.
		Adjudication: Both translators and the reviewer discussed the translations and suggestions and decided for a final translation.
		Documentation: Draft translations, exchange of comments between the translators and decisions for a final translation was documented.
2	Assessment of comprehensibility by two rounds of cognitive interviews	Convenience sample including nurses and physicians of a Comprehensive Cancer Center in Hamburg, Germany as well as psychologists of the out-patient clinic for Psycho-oncology at the University Medical Center Hamburg – Eppendorf (UKE).
3	Psychometric evaluation by secondary analysis of cross-sectional data	The sample includes all physicians and nursed working in one of three departments of a Comprehensive Cancer Center in Hamburg, Germany

### Measure

The IcanSDM was developed based on empirical and theoretical work as well as existing literature [28, 35, 43]. Additionally, the design of the scale was guided by the Ottawa Model of Research Use [43] and the Theory of Planned Behaviour [29]. Items of the IcanSDM can be rated on an analogue scale ranging from 0 (“strongly disagree”) to 10 (“strongly agree”) [35]. In a first version, the original IcanSDM consisted of eleven items. Two of these items have shown to reduce internal consistency, one item was not understood by participants in a qualitative item analysis and also has shown to reduce internal consistency. These three items were excluded for further development and validation of the scale [35]. Items were developed in French and translated into English by a translator for international publication. For the English items of the 8-item scale, see Table 4 of the Results section. The authors of the original IcanSDM are the only ones who provided psychometric data for the IcanSDM up to now. Their analysis revealed a Cronbach’s α of above 0.63 and a trend to sensitivity to change [35]. Additionally, they assumed that the measure has a one-dimensional structure, but the factorial structure of the IcanSDM has not been evaluated up to now. Giguere et al. [35] recommend to calculate a sum score for all items. Higher scores indicate stronger perception of barriers, which might be associated with lower ability to adopt SDM.

### Translation

To be published in an international journal, the original French IcanSDM was translated into English by an official translator. We translated the English IcanSDM into German. Thereby we used the team translation protocol TRAPD (Translation, Review, Adjudication, Pretesting and Documentation [44–47]). As a first step, two team members (AL, NE, cp. list of abbreviations), independently translated the English IcanSDM including the response scale, the introduction and all eight items into German. In a second step, a third team member (IS) reviewed the translations of AL and NE. She decided for one of the two versions or suggested a third version. Additionally, another team member (SZ, cp. list of abbreviations) reviewed the translations by AL, NE, and IS and made additional suggestions for single items. All team members were proficient in English and German. In a final discussion, AL, NE, and IS reached a consensus on a final German IcanSDM version to be further tested for comprehensibility.

### Assessment of comprehensibility

To measure if the IcanSDM items reflect the construct of perception of SDM barriers, we assessed content validity. Thereby we followed COSMIN criteria (Consensus-based standards for the selection of health measurement instruments) [48, 49], which define content validity as relevance, comprehensiveness, and comprehensibility of the measures items and scales. When translating an existing measure, it is most interesting to assess comprehensibility. Thus, we aimed to evaluate if items are worded appropriately and understood by HCPs as intended by the developers of the measure..

To assess comprehensibility, we executed two rounds of cognitive interviews. Items, which showed less comprehensibility in the first round of cognitive interviews were modified by the study team and again tested in a second round. We recruited a convenience sample of HCPs including nurses, physicians, and psychologists who worked at a Comprehensive Cancer Center in Hamburg, Germany. We excluded HCPs who worked in clinics which take part in the SDM implementation study, where the IcanSDM was planned to be applied. We invited physicians and nurses by sending an e-mail to head physicians and leading nurses of clinics within the Comprehensive Cancer Center including the request to forward the invitation to physicians and nurses of the clinics. Psychologists were recruited via the out-patient clinic for Psycho-oncology at the University Medical Center Hamburg – Eppendorf (UKE). AL and PH conducted the interviews, which took place in a seminar room of the Department of Medical Psychology at the University Medical Center Hamburg – Eppendorf (UKE). We developed an interview guide, including verbal probing and paraphrasing [50, 51]. Cognitive interviews were audio-recorded and transcribed verbatim. In a first round of cognitive interviews, participants were asked for their comprehension of the response scale, the introduction as well as one translated version for item 1 to item 7 and two translated versions for item 8. Afterwards, we extracted participants’ comments from the transcripts and discussed them within the study team (AL, PH, IS). Items of the German IcanSDM, which were not well understood by participants, were adapted accordingly. These revised items were tested in a second round of cognitive interviews. Afterwards, we again had to discuss translation and further adaption of one item (item 8). For this step, we involved one of the authors of the original IcanSDM measure (AG) and discussed the meaning and the background literature of item 8. As a consequence of our discussion, AG revised item 8 of the original French and English IcanSDM to increase comprehensibility by participants. We again translated this revised item 8 using the same approach as described above.

We used SPSS (IBM SPSS Statistics, Version 23) to calculate descriptive statistics of participants’ demographic characteristics.

### Psychometric evaluation

#### Data collection

Within the scope of a SDM implementation study [22], we conducted a secondary analysis of cross-sectional data. The IcanSDM was one of several measures [52–54] within one survey (27 items), which also assessed demographic characteristics (5 items).

We included all physicians and nurses working at one of the three departments of one Comprehensive Cancer Center in Germany, which participated in the SDM implementation study [22]. We expected physicians and nurses with diverse demographic characteristics including different sex, age and work experience taking part in this study. Physicians and nurses received the survey either (1) personally by a study team member, (2) via supervising nurses, or (3) via mail. After filling out the survey, participants had the options to either (1) personally hand the survey over to a study team member or (2) to send it back via mail. For quality control, we entered 20% of the data double and blinded into SPSS (IBM SPSS Statistics, Version 23).

#### Data analysis

To assess acceptance of the IcanSDM, we evaluated the completion rate and calculated missing data frequencies per item, per case, and for the overall measure. For further psychometric evaluation, we excluded cases with more than 30% of IcanSDM items missing (3 items and more) [55]. Missing data for the other cases were replaced by means of the single items. We calculated descriptive statistics for demographic characteristics of participants.

For item analysis, we examined response distribution end evaluated floor and ceiling effects [56]. For floor and ceiling effects we analysed item difficulties [57, 58] as well as the skewness of response distribution [59]. Additionally we calculated item means and standard deviations, corrected item-total correlations [60, 61], and inter-item correlations [60, 61].

To test assumptions for performing a factor analysis, Kaiser-Meyer-Olkin (KMO) measure of sampling adequacy and Bartlett’s test of sphericity were calculated [57, 60]. Analysis of the factorial structure of the IcanSDM has an exploratory character based on assumptions of Giguere et al. They calculated psychometric analysis of the French IcanSDM based on an assumed one-factorial structure [35]. Accordingly, we a priori hypothesized a one-factorial structure of the German IcanSDM. A confirmatory factor analysis (CFA) with Maximum Likelihood Estimates and one factor was applied for the whole data set (model 1). During factor analysis we modified this model stepwise (model 2 and model 3). For the interpretation of factor loadings and definition of cut-offs for suitable factor models, different criteria exist [59, 62–64]. Therefore, interpretation of model quality might differ depending on the underlying theory and applied cut-off values. We decided to use the established and less conservative criterion of .40 for sample sizes above *n* = 200 as a cut-off for acceptable factor loadings [63, 64]. We calculated global goodness of fit indices: discrepancy chi-squared statistic (Chi^2^), degree of freedom (df), normed chi-squared statistic (Chi^2^/df), Comparative Fit Index (CFI), Tucker-Lewis Index (TLI), Root Mean Square Error of Approximation (RMSEA), as well as the Akaike Information Criterion (AIC) and Parsimonious Normed Fit Index (PNFI), which hep to analyse model complexity. We used established criteria to interpret the estimated model fits [63, 65–67], see Table 2.

**Table 2:** Psychometric analysis conducted

Psychometric measure	Criteria
Kaiser-Meyer-Olkin (KMO) measure of sampling adequacy and Bartlett’s test of sphericity	These tests ensure that correlations between variables can be accounted for by a smaller set of factors [60]. KMO value should be higher than .05 and Bartlett’s test value should be less than .05 to fulfil the criteria for calculating a factor analysis [57, 60].
Normed chi-squared statistic (Chi^2^/df)	Chi^2^/df is an indicator for model fit, dependent on sample size and should be as small as possible. A ratio between 2 and 3 indicate a good data fit [67].
Comparative fit indexes (CFI)	CFIs is an indicator for model fit. It ranges from 0 to 1 and higher values indicate better fit. Values above .95 indicate a good model fit [65, 68].
Tucker-Lewis Index (TLI)	TLI is an indicator for model fit. It corrects for complexity of the model and is sensitive to small sample sizes. Values above .95 indicate good fit [66].
Root mean square error of approximation (RMSEA)	RMSEA is an absolute index which describes closeness to fit. Values below .05 indicate a good fit, values between .05 and .08 indicate an adequate fit, values between .08 and 1 indicate a moderate fit and values above 1 are unacceptable [69].
Akaike Information Criterion (AIC)	AIC is a parsimony model fit index. It can be used to compare fit of competing models with smaller values indicating better fit [65, 67].
Parsimonious Normed Fit Index (PNFI)	PNFI is a parsimony model fit index. It ranges between 0 and 1 and higher values indicate a more parsimonious fit [67]. No threshold levels are recommended and it has to be analysed in combination with other goodness of fit indices [65].
Analysis of frequencies for item response distributions	Floor and ceiling effects were assumed if more than 15% of participants choose the lowest or highest possible score [56]. For analogue scales, no cut-off values exist. According to Bortz & Döring, items with difficulties below 20% show a floor effect, items with difficulties above 80% show a ceiling effect. Additionally, a skewness below − 2 indicates a floor effect, a skewness above + 2 indicates a ceiling effect [59].
Corrected item-total correlations	If items correlate with the total score of above .30, they measure the same underlying concept. Items with lower correlations should be removed because they do not add exploratory power to the measure [60, 61].
Item difficulties	Item difficulties are calculated by dividing item means by the maximal value of the answer range (0–10) and multiplying it with 100. Item difficulty should be near to 50%, and items should not differ much in their difficulty level [57].
Inter-item correlations	Inter-item correlations ensure association between items. High inter-item correlations of above .80 indicate that items ask the same questions and might be redundant [60, 61].
Cronbach’s α	Cronbach’s α is a measure for reliability and internal consistency. A value of at least .70 is acceptable and higher coefficients indicate a more stable measure [57, 60, 70].

Note: This table has been adapted from Lindig et al. [53]

Because factor loadings and model fit indices for the one-factor model were not satisfying, we applied two alternative models (model 2 and model 3) to the data based on analysis of modification indices and content of specific items (see Results section).

We assessed internal consistency by Cronbach’s alpha coefficient (α) [57, 61, 70]. Since this was a secondary analysis of cross-sectional data, several psychometric parameters like convergent or divergent validity were not analysable.

For a detailed overview on performed data analysis, see Table 2.

For analysis of demographic data, completion rate and item analysis we used SPSS (IBM SPSS Statistics, Version 23). For CFA and calculation of model fit indices we used Amos (IBM SPSS Amos 22.0.0).

## Results

### Translation

Both translators (AL and NE) and the reviewer (IS) came to similar translations of the IcanSDM. Only slight differences in sentence structure or single words without differences in meaning were found. SZ reviewed all translations and added comments and suggestions for items 2, 4, and 8. During the following team discussion, we reached consensus for translation of all items, the response scale, and the survey introduction.

### Assessment of comprehensibility

We conducted cognitive interviews with *n* = 11 participants to test the German IcanSDM for comprehensibility. For demographic data of participants see Table 3.

**Table 3:** Demographic data of participants of cognitive interviews (*n* = 11)

	Frequencies for n = 11
Age	
< 30 years	1
31–40 years	3
41–50 years	3
> 50 years	4
Sex	
Female	9
Male	2
Profession^a^	
Nurse	8
Physician	2
Psychooncologist	3
Work experience in health care	
> 5 years	3
5–10 years	4
11–20 years	3
> 20 years	1

^a^ multiple answers possible

The first round of cognitive interviews (*n* = 7) revealed that items 1, 2, 3, 4, 7, and the response scale were well understood. We found lower comprehensibility for the survey instruction, item 5, 6 and 8. The survey instruction and the three items were tested again in a second round of cognitive interviews (*n* = 4).

When filling out the measure, some participants of the first round of cognitive interviews did not refer to their own opinion and think about their current work place. We concluded that the survey instruction might not be precise enough. The revised survey instruction was well understood by all participants of the second round of cognitive interviews.

For item 5 (‘Shared decision making takes up too many resources.’), participants of the first round of cognitive interviews suggested to add ‘e.g. time, staff’ as examples for the term ‘resources’ to increase comprehensibility of this term. After changing the item accordingly, it was well understood by all participants of the second round of cognitive interviews.

For item 6 (‘Shared decision making is inconsistent with clinical practice guidelines.’), some participants of the first round of cognitive interviews were not sure about the meaning of the term ‘clinical practice guidelines’. For the second round of cognitive interviews, we therefor tested item 6 again as well as an alternative version with a slight change in wording. Since the original translation of item 6 was now understood best, we decided to use this translation for the final measure.

For nearly all participants of the first round of cognitive interviews, the meaning of item 8 (‘The shared decision-making process highlights the uncertainty associated with interventions. This could affect treatment adherence.’) was not clear. Especially the term ‘treatment adherence’ was not familiar for participating nurses and the sentence structure was confusing for some participants. Some participants suggested to rather use the term ‘compliance’ because it is more commonly used in daily practice. Nevertheless, we discussed that ‘treatment adherence’ is the less stigmatic term. We therefor rephrased the item and tested two new versions in the second round of cognitive interviews. In the first version of revised item 8 we used the German phrase ‘Behandlungstreue’, which seemed to be better understood compared to ‘Behandlungsadhärenz’ (both translate into ‘treatment adherence’ in English). In the second version of item 8 we used the phrase ‘compliance’ according to suggestions of some participants. Participants of the second round of cognitive interviews still differ in their comprehension of this item. We had to conclude that item 8 could not be translated successfully indicating reduced comprehensibility according to COSMIN criteria [48]. For the subsequent revision in collaboration with AG, we changed the structure of the English and French item and left out the term ‘treatment adherence’: ‘During shared decision making, the patient becomes aware of the uncertainty associated with interventions and might become confused’. After translation by the study team, we reached consensus on a final version of item 8. This item was not further tested in cognitive interviews.

For the original version of the IcanSDM and the versions used for both rounds of cognitive interviews, see Table 4. For the final German IcanSDM measure, see Additional file 1.

**Table 4:** English version of the items and German translation

	Original version	Versions used for the first round of cognitive interviews	Versions used for the second round of cognitive interviews
Response scale	strongly disagree strongly agree	Stimme überhaupt nicht zu. Stimme völlig zu.	/ /
Introduction	Please indicate how much you agree or disagree with each of the following statements relative to shared decision making. There is no right or wrong answer.	Bitte geben Sie an, wie stark Sie jeder der folgenden Aussagen zur partizipativen Entscheidungsfindung zustimmen. Es gibt keine richtige oder falsche Antwort.	Bitte geben Sie durch ein Kreuz auf der Linie an, wie stark Sie jeder der folgenden Aussagen zustimmen. Geben sie Ihre persönliche Meinung an und beziehen Sie sich auf Ihren aktuellen Arbeitsplatz.
Item 1	Shared decision - making results in longer clinical encounters.	Partizipative Entscheidungsfindung führt zu längeren Gesprächen mit Patientinnen und Patienten.	/
Item 2	Patients often prefer that the clinician make the decision.	Patientinnen und Patienten finden es häufig besser, dass der Arzt / die Ärztin die Entscheidung trifft.	/
Item 3	Shared decision making does not apply to all patients, nor does it apply to all clinical situations.	Partizipative Entscheidungsfindung kann man weder bei allen Patientinnen und Patienten noch in allen klinischen Situationen anwenden.	/
Item 4	Communicating scientific data to patients is too complex.	Es ist zu aufwendig, Patientinnen und Patienten wissenschaftliche Daten zu vermitteln.	/
Item 5	Shared decision making takes up too many resources.	Partizipative Entscheidungsfindung beansprucht zu viele Ressourcen.	Partizipative Entscheidungsfindung beansprucht zu viele Ressourcen (z.B. Zeit, Personal).
Item 6	Shared decision making is inconsistent with clinical practice guidelines.	Partizipative Entscheidungsfindung ist nicht mit klinischen Leitlinien vereinbar.	Partizipative Entscheidungsfindung ist nicht mit klinischen Leitlinien vereinbar.
			Partizipative Entscheidungsfindung ist nicht mit den Vorgaben in klinischen Leitlinien vereinbar.
Item 7	Shared decision making is just a passing trend.	Partizipative Entscheidungsfindung ist nur ein vorübergehender Trend.	/
Item 8	The shared decision-making process highlights the uncertainty associated with interventions. This could affect treatment adherence.	Der Prozess der partizipativen Entscheidungsfindung beleuchtet die Unsicherheit, die mit Behandlungen verbunden ist. Dies könnte die Behandlungsadhärenz beeinflussen.	Partizipative Entscheidungsfindung macht vorhandene Unsicherheiten in Bezug auf Behandlungen deutlich. Dies könnte die Behandlungstreue beeinflussen.
		Der Prozess der partizipativen Entscheidungsfindung beleuchtet die Unsicherheit, die mit Behandlungen verbunden ist. Dies könnte die Behandlungstreue beeinflussen.	Partizipative Entscheidungsfindung macht vorhandene Unsicherheiten in Bezug auf Behandlungen deutlich. Dies könnte die Compliance beeinflussen.

Some nurses reported during cognitive interviews, that the term “shared decision-making” (German: “Partizipative Entscheidungsfindung”) was new to them and they had not heard about the concept of SDM before. Thus, a definition of SDM was provided in the introduction of the survey.

### Psychometric evaluation

#### Missing data analysis and analysis of acceptance

We collected data of 243 HCPs. For the whole data set, 20 missing values could be observed. Resulting completion rates per item ranged between 97.95% (five missing values for item 6 and 7) and 99.59% (one missing value for item 1, 3, and 5) (see Table 6). Missing values could be observed for 11 cases (eight cases with one missing value, two cases with two missing values, and one case for which all 8 items were missing). Accordingly, 98.97% of all items over all participants were answered (1924 of 1944 data points) and 95.47% of the respondents completely filled out the measure. One case (0.4% of all cases) had to be excluded from further analysis because all IcanSDM items were missing [55]. For the other cases, 0.62% of all items (11 of 1936 data points) were missing and replaced by item means. Therefore, data of 242 HCPs were included in the psychometric analysis.

#### Sample characteristics

Table 5 gives an overview of participants’ demographic characteristics. The majority of the 242 HCPs were between 31 and 40 years old (36.0%), female (71.1%), were nurses (57.0%), and had work experience of less than 5 years (43.8%).

**Table 5:** Demographic characteristics of participants (*n* = 242 healthcare professionals)

	N (%)
Age	
< 30 years	76 (31.4)
31–40 years	87 (36.0)
41–50 years	44 (18.2)
> 50 years	29 (12.0)
Missings	6 (2.5)
Sex	
Female	172 (71.1)
Male	60 (24.8)
Different sex or preferred not to answer this question	4 (1.7)
Missings	6 (2.5)
Profession	
Nurse	138 (57.0)
Physician	99 (40.9)
Junior physician	70 (28.9)
Senior physician	29 (12.0)
Missings	5 (2.1)
Work experience	
< 5 years	106 (43.8)
5–10 years	50 (20.7)
11–20 years	43 (17.8)
> 20 years	34 (14.0)
Missings	9 (3.7)

#### Analysis of the IcanSDM items

**Fig. 1:**
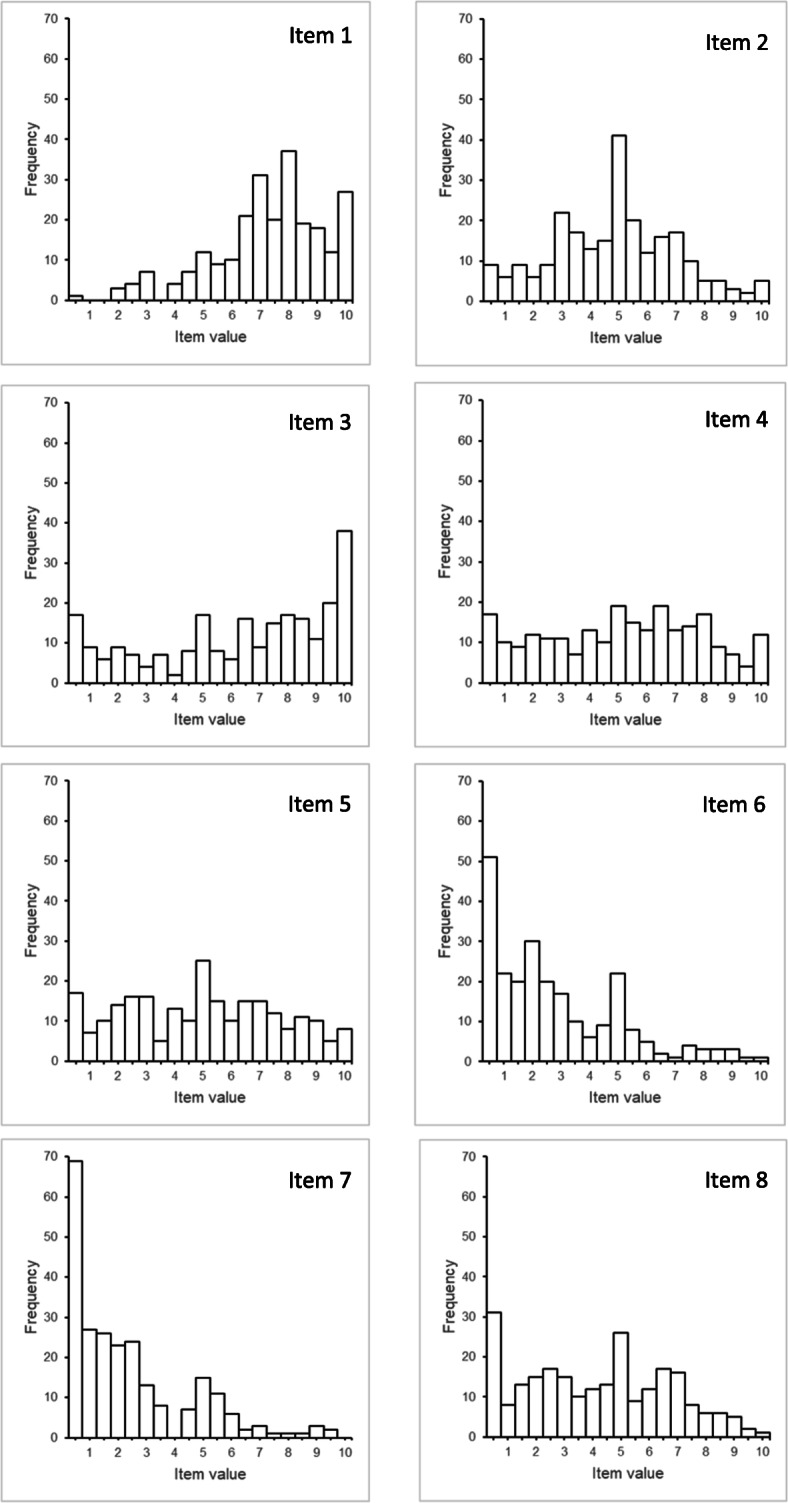
Response distribution for the IcanSDM items (*n* = 242 healthcare professionals). Data were clustered in steps of 0.5

Response distributions for all items are presented in Fig. 1. Observation of response distribution showed a trend to floor effects for items 6 and 7 and a slight trend to a ceiling effect for item 3. Table 6 shows means and standard deviations, skewness, item difficulties, acceptance (completion rates), and item discrimination (corrected item-total correlations) of the eight items. Means ranged between 2.16 (item 7) and 7.09 (item 1) on a scale from 0 to 10. Accordingly, item difficulties ranged from 21.63 (item 7) to 70.85 (item 1). Skewness for the items are acceptable since values are between −.797 (item 1) and 1.25 (item 7) [59]. Skewness and item difficulties indicate that no floor or ceiling effects can be observed for our data [57–59]. Corrected item-total correlations ranged from .200 (item 1) to .475 (item 4), and inter-item correlations from .005 (item 3 / item 6) to .412 (item 3 / item 5, see Table 7).

**Table 6:** Means, standard deviation, skewness, item difficulty, acceptance and item discrimination of the German IcanSDM (*n* = 242 healthcare professionals)

	Items	Mean (SD)	Skewness	Item difficulty	Acceptance (Completion rate in %)	Item discrimination (corrected item-total correlation)
1	Shared decision-making results in longer clinical encounters.	7.08 (1.97)	−.797	70.85	99.59	.200
2	Patients often prefer that the clinician makes the decision.	4.64 (2.21)	.071	46.44	99.18	.347
3	Shared decision-making does not apply to all patients, nor does it apply to all clinical situations.	6.13 (3.16)	−.5	61.25	99.59	.257
4	Communicating scientific data to patients is too complex.	4.94 (2.76)	−.106	49.39	99.18	.475
5	Shared decision-making takes up too many resources.	4.75 (2.72)	.046	47.47	99.59	.459
6	Shared decision-making is inconsistent with clinical practice guidelines.	2.58 (2.25)	1.00	25.79	97.94	.256
7	Shared decision-making is just a passing trend.	2.16 (2.16)	1.25	21.63	97.94	.389
8	During shared decision-making, the patient becomes aware of the uncertainty associated with interventions and might become confused.	3.97 (2.53)	.152	39.70	98.77	.383

**Table 7:** Inter-item correlation matrix for the German IcanSDM (*n* = 242 healthcare professionals).

	Item 1	Item 2	Item 3	Item 4	Item 5	Item 6	Item 7	Item 8
Item 1	1.000	.165	.128	.158	.266	−.041	.005	.076
Item 2	.166	1.000	.132	.171	.252	.084	.282	.277
Item 3	.128	.132	1.000	.290	.103	.-005	.055	.282
Item 4	.158	.171	.290	1.000	.412	.245	.228	.234
Item 5	.266	.252	.103	.412	1.000	.235	.307	.201
Item 6	−.041	.084	−.005	.245	.235	1.000	.392	.119
Item 7	.005	.282	.055	.228	.307	.392	1.000	.251
Item 8	.076	.277	.282	.234	.201	.119	.251	1.000

#### Factor analysis

Assumptions for factor analysis were fulfilled [48, 57, 60]. KMO measure was .698 and Bartlett’s test of sphericity resulted in X^2^ = 247.43, *p* < .001 [57, 60].

**Fig. 2:**
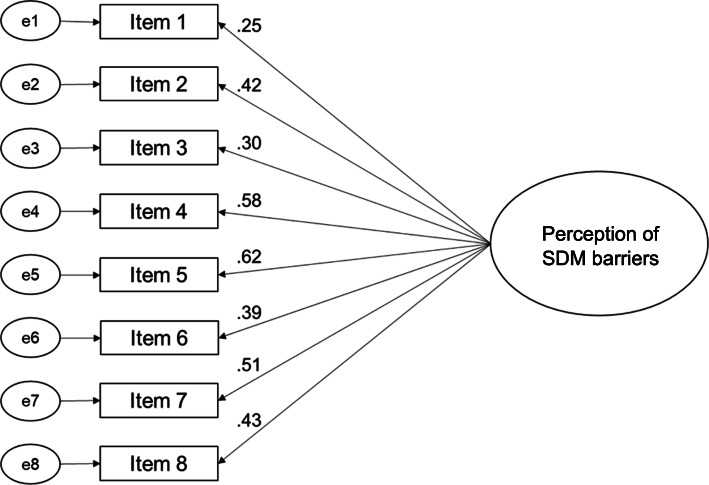
Confirmatory factor analysis model for a one-factorial structure (model 1)

For model 1, we assumed a one-factorial structure without correlations between items based on the evaluation of the original measure [35]. CFA for this model showed standardized factor loadings between .25 (item 1) and .62 (item 5) with three items showing factor loadings below .40 (items 1, 3, and 6; see Fig. 2). This indicated that some items did not fit to the predefined factor [59, 63, 64]. A Chi^2^/df of above 3.0, CFI, TLI of below .95, and RMSEA of above .1 did not meet cut-offs, indicating that the one-factor model did not fit to the data [65–67, 69] (see Table 8). Additionally, observed modification indices between item 3 and item 8 (8.42) as well as item 6 and item 7 (16.85) were high. This indicates a model fit improvement if correlations between these items are allowed. Thus, correlations between items 3 and 8 as well as items 6 and 7 could be postulated. We also analysed these items on the content level. Item 3 and item 8 both address the barrier that SDM might not be suitable for (all) patients in all clinical situations. Compared to all other items, which address the interaction between HCP and patient, item 6 and item 7 both target the macro level. We therefore calculated an alternative model with correlated residuals between item 3 and item 8 as well as between item 6 and item 7 (model 2).

**Table 8:** Fit indices of three calculated models for factor analysis of the German IcanSDM (*n* = 242 healthcare professionals)

	Chi^2 a^	df ^b^	Chi^2^/df ^c^	CFI ^d^	TLI ^e^	RMSEA ^f^	AIC ^g^	PNFI ^h^
Model 1	72.48*	20	3.62	.765	.671	.104	120.48	.508
Model 2	45.51	18	2.53	.878	.808	.080	97.51	.526
Model 3	53.41	19	2.81	.846	.773	.087	103.41	.534

Notes: Model 1 is a one-factor model with no correlations between items. Model 2 is a one-factor model with correlations between items 3 and 8 as well as items 6 and 7. Model 3 is a two-factor model with factor 1 including items 1 to 5 and 8 and factor 2 including item 6 and 7. All models were calculated for the whole data set (n = 242)

^a^ Discrepancy chi-squared statistic, ^b^ Degrees of freedom, ^c^ Normed chi-squared statistic,

^d^ Comparative fit indexes, ^e^ Tucker-Lewis Index, ^f^ Root mean square error of approximation, ^g^ Akaike Information Criterion, ^h^ Parsimonious Normed Fit Index (PNFI). * *p* = .000

**Fig. 3:**
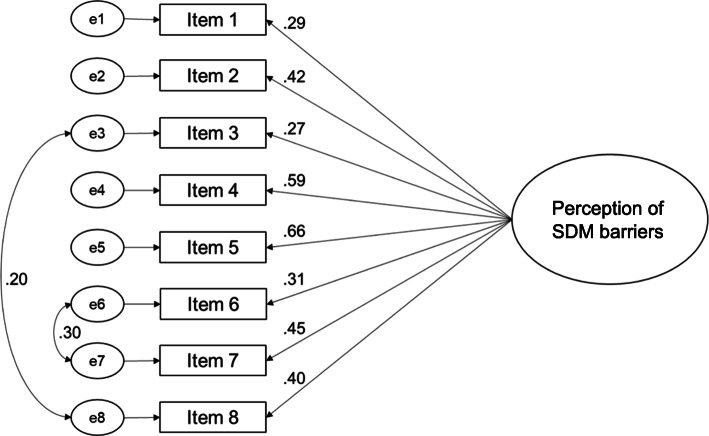
Confirmatory factor analysis model for a one-factorial structure with correlations between items 3 and 8 as well as items 6 and 7 (model 2)

Model 2 came along with factor loadings between .29 (item 1) and .66 (item 5) with three items showing factor loadings below .40 (items 1, 3 and 6; see Fig. 3). Model fits for model 2 were better than for model 1 (see Table 8). Values for Chi^2^/df and RMSEA were acceptable, values for CFI and TLI were still not acceptable [65–67, 69]. Because of its unacceptable factor loadings and model fits, we had to conclude that model 2 also does not fit to the data. Since item 6 and item 7 fundamentally differ from all other items (they target the macro level), we tested another model with two factors (model 3). Factor 1 included items 1 to 5 and item 8, and factor 2 included items 6 and 7. We assumed a correlation between both factors.

**Fig. 4:**
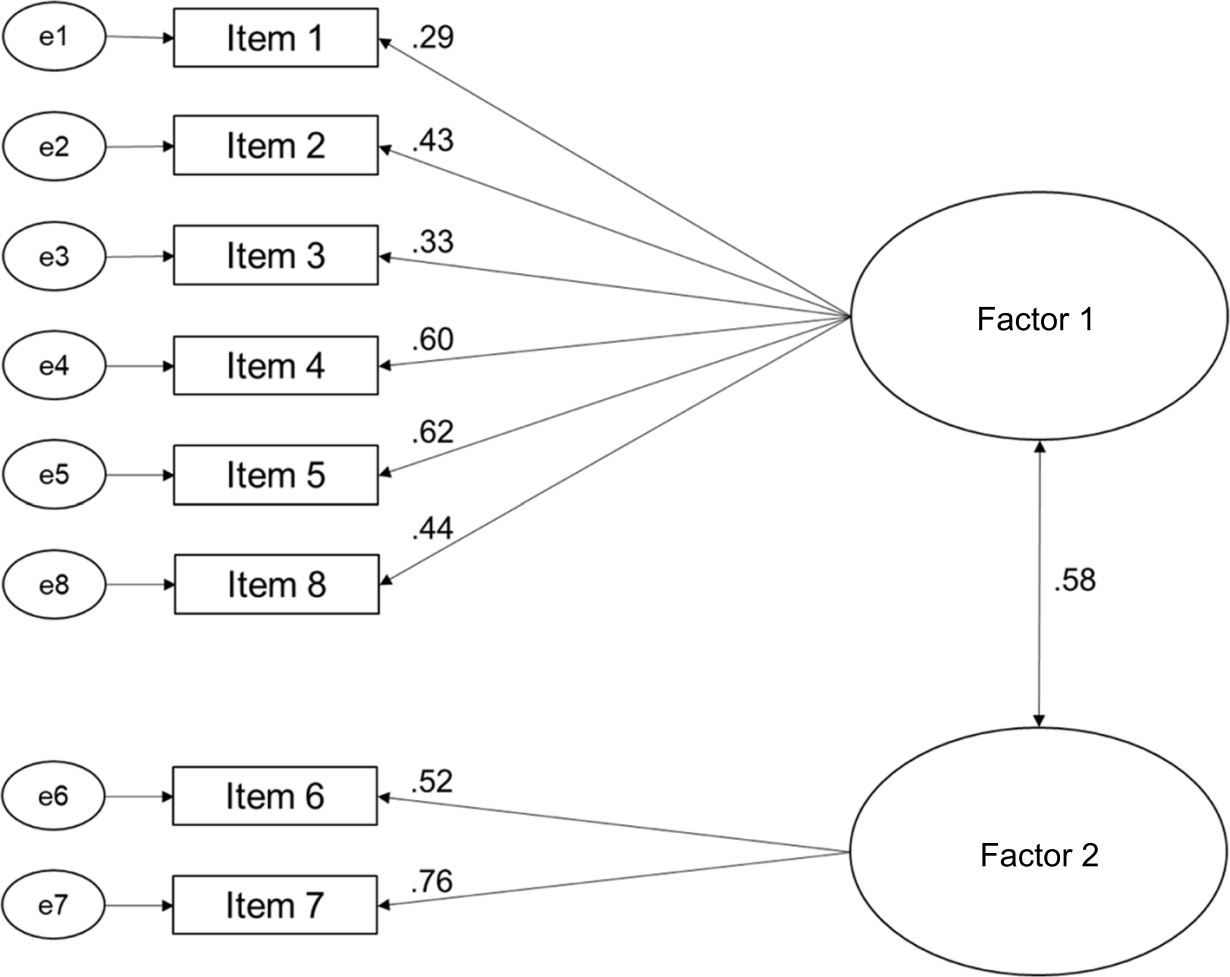
Confirmatory factor analysis model for a two-factorial structure (model 3)

Model 3 came along with factor loadings between .29 (item 1) and .76 (item 8) with two items showing factor loadings below .40 (items 1 and 3; see Fig. 4). Correlation between both factors was .58. Model fits for model 3 were comparable to model fits of model 2, but factor loadings were better for model 3 [59, 63, 64] (see Table 8).

#### Analysis of internal consistency

Cronbach’s α is .651 for the one-factor models 1 and 2. For model 3 with two factors, Cronbach’s α for factor 1 is .613 and Cronbach’s α for factor 2 is .563.

## Discussion

The IcanSDM is a brief measure to assess HCPs’ perceptions of SDM barriers. It was recently developed and evaluated in Canada for French speaking physicians and translated into English [35]. Aim of this study was to translate and adapt the English IcanSDM to be used by a German speaking population and evaluate its psychometric properties.

### Translation and psychometric evaluation

Study team members involved in translation reached consensus for the translation of the IcanSDM. While items 1 to 7 and the survey instruction were well understood by all participants of cognitive interviews, item 8 had to be reworded. Comprehensibility of the final item 8 was not further tested in cognitive interviews, thus content validity of this item remained unclear. During cognitive interviews, we noticed that some participants, especially nurses, were less familiar with the concept of SDM than others. This is notable since SDM is promoted by the German government and research for many years [71–74]. Several studies could show that HCPs often lack a full conceptual understanding of SDM while at the same time having a positive attitude towards SDM [33, 75–79]. But it has to be kept in mind that limited knowledge about SDM might also be a barrier to perform SDM [79]. In terms of cross-cultural translation of a SDM measure, this underlines the importance of assessing knowledge about the construct and specific terms of the measure in the target language and compare it with the original language [38]. For filling out the IcanSDM it is crucial to have an idea about the concept of SDM. Therefore, we would recommend to provide a definition of SDM in the introduction of the measure.

In the following CFA, we could not confirm the a priori hypothesized one-factorial structure for the German IcanSDM (model 1) or an alternative model with correlations between item 3 and item 8 as well as item 6 and item 7 (model 2). A model with two factors (model 3) showed a correlation of .58 between both factors, which indicates that the factors are associated but capture different aspects of SDM barriers. This is congruent with results of Giguere et al. [35], who found a shift in item response distribution towards a more positive attitude after training for all items except for items 6 and 7. The authors concluded that training might have less impact on rating of these items. This underlines our assumption that item 6 and 7 rather cover SDM barriers on the macro level, where perceived behavioural control might be reduced compared to other items. Nevertheless, model 3 did not show satisfying model fits [59, 63, 64]. To conclude, we could not provide a valid model for the German IcanSDM at this point of analysis. This might be due to an insufficient cross-cultural translation process [38]. There might be differences in the concepts measured by the IcanSDM in the original and the target language. Additionally, we may missed to address differences in the German and Canadian health care settings. Furthermore, we had to deal with some time and financial constraints because the IcanSDM was planned to be used in an SDM implementation study [22]. For practical reasons, we therefore did not pretest the measure. A flawed cross-cultural adaptation process might result in reduced comparability of the Canadian and German IcanSDM [38, 39]. Future studies should explore all aspects of cross-cultural validity and the needs to adapt the measurement for the cultural context of the German health care system. Thereby, it should be assessed whether the IcanSDM items are relevant and comprehensive to assess SDM barriers in Germany and whether results are comparable with samples from other countries and of other languages.

The German IcanSDM was found to be well-accepted by study participants. Items, which had low factor loadings in the factor analysis also showed conspicuous characteristics in item analysis. Observed floor and ceiling effects indicated that items 6 and 7 might not be perceived as barriers for SDM in the German healthcare system and item 3 might be phrased too conservative. Corrected item-total correlations of items 1, 3 and 6 indicated that these items do not measure the underlying concept [60, 61]. Criteria for good item difficulties were not met for items 1, 6, 7, and 8 [57]. Inter-item correlations indicated that all items add additional information but are less connected to each other [60, 61]. This also results in low values for internal consistency which are comparable to the original scale [57, 60, 70]. Nevertheless, since no valid model could be found for the German IcanSDM, values for internal consistency should be interpreted with caution.

### Use of the IcanSDM as a measure for perception of SDM barriers by HCPs

The authors of the French IcanSDM hypothesized that clinicians’ perception of SDM barriers could indicate their perceived ability to adopt SDM. Giguere et al. [35] evaluated the psychometric properties of the IcanSDM in a small sample and described a trend towards change sensitivity but also conspicuous item characteristics and suboptimal low values for Cronbach’s α. In our study, we replicated these findings with an adequate sample size, indicating that the different items may not measure the same underlying construct. This might be a result of the fact that the items target SDM barriers on different levels (e.g. attitude, organizational level, social norms). All in all, the results indicate that the construct that the IcanSDM claims to measure needs revision. Thus, this limits interpretation of findings for the original scale and comparison with the German IcanSDM.

Therefore, we recommend to further evaluate and revise the underlying construct of the original IcanSDM scale as well as the German IcanSDM scale. It should also be considered to add items to assess perceived behavioural control upon adoption of SDM as well as to add more items which describe barriers at the macro level. Additionally, during the development of the measure, the authors of the original IcanSDM deleted three items due to their negative effects on internal consistency and comprehensiveness of the measure. These items might be relevant for the German IcanSDM and should be considered for use in the German IcanSDM. To use the revised original IcanSDM in German, it should go through a systematic cross-cultural translation process [39]. Afterwards, the revised German IcanSDM scale should be assessed with a new sample of HCPs. Also, comparisons with other measures to test for convergent and divergent validity might help to evaluate and improve the quality of the scale.

In a next step, it is important to assess if the IcanSDM can reflect HCPs’ perceived ability to adopt SDM and therefore predict future behaviour of HCPs. Thereby, results of the French and German IcanSDM should be compared with actual and objectively measured SDM uptake by HCPs. Our SDM implementation study is a first step into this direction [22].

Furthermore, knowledge of HCPs’ perceptions of SDM barriers can be helpful to prospectively plan a future SDM implementation study. IcanSDM results can be used to select suitable settings where SDM implementation might be most effective or to specifically address mostly perceived barriers.

### Strengths of this study

We provided the first measure, which evaluated HCPs perception of SDM barriers in German language. The IcanSDM can be used in health services research and implementation studies. We followed an established translation procedure based on recommendations for cross-cultural translation studies. Additionally, we analysed comprehensibility of the items. For psychometric analysis we included physicians and nurses with diverse demographic characteristics. The sample size was adequate for conducting psychometric analysis.

### Limitations of this study

This study has seven limitations. Since this was a secondary analysis of cross-sectional data, psychometric parameters like convergent or divergent validity were not analysable. Second, data collection was limited to three departments of one Comprehensive Cancer Centre in Germany. Because we did not hand over the survey to all participants personally, we cannot control that all eligible physicians and nurses received the survey. Furthermore, a self-selection bias of participants who are interested in the topic cannot be excluded. The psychometric properties of the IcanSDM are therefore not automatically transferable across translations and other settings. To make assumptions about generalizability, further validation in other settings is necessary. Third, we translated and adapted the English version of the French IcanSDM. The English IcanSDM was translated for publication by an official translator and was not reviewed in a cross-cultural validation study. Thus, we did not translate and adapt the IcanSDM from its original language. Fourth, we had to assess a convenience sampling approach instead of a purposive sampling approach in cognitive interviews because it was difficult to recruit participants, especially physicians. Fifth, the final item 8 was not further tested in cognitive interviews, thus comprehensibility of this item remained unclear. Sixth, for handling missing data, we used mean replacement, which can lead to biased estimates. However, because the amount of missing data in our data set is very low (0.62%), the probability of a potential bias should be quite low. Seventh, the results of the developmental process and psychometric evaluation of the French IcanSDM are not yet published in a peer-reviewed journal. Since we conducted the first translation and psychometric evaluation of the French IcanSDM, other data on IcanSDM translations are missing.

## Conclusion

HCPs’ perceptions of SDM barriers should be measured with valid and reliable measures. We provide the first German measure for assessing this construct. The German IcanSDM is a brief measure with good acceptance. However, when assessing in a German hospital setting, we found unsatisfying psychometric properties, which were comparable to results of the original scale. Therefore, the German as well as the original IcanSDM need further revision and evaluation, especially regarding content validity. In a next step, predictive validity of the French and German IcanSDM should be evaluated. If the IcanSDM is further developed and revised successfully, it has the potential to assess perception of SDM barriers as predictors for SDM uptake in SDM implementation studies in German and international healthcare settings.

## Supplementary Information

**Additional file 1.** IcanSDM – German version.

## Data Availability

The dataset collected and analyzed during this study is available from the corresponding author on reasonable request and after consultation of the Ethics Committee of the Medical Association Hamburg.

## Abbreviations

AG: Anik Giguere
AIC: Akaike Information Criterion
AL: Anja Lindig
AMOS: Analysis of Moment Structure, Statistical Package for the Social Sciences, International Business Machines Corporation
Chi^2^: Discrepancy chi-squared statistic
Chi2/df: Normed chi-squared statistic
CFI: Confirmatory fit index
Df: Degree of freedom
EC: Eva Christalle
HCP: Healthcare professional
IS: Isabelle Scholl
KMO: Kaiser-Meyer-Olkin criterion
MH: Martin Härter
NE: Nina Elpers
OK: Olaf von dem Knesebeck
PH: Pola Hahlweg
PNFI: Parsimonious Normed Fit Index
RMSEA: Root mean square error of approximation
SDM: Shared decision-making
SPSS: Statistical Package for the Social Sciences, International Business Machines Corporation
SZ: Stefan Zeh
TLI: Tucker-Lewis Index
TRAPD: Translation, Review, Adjudication, Pretesting, Documentation
