# Supplementary material for: Translation and psychometric evaluation of the German version of the IcanSDM measure – a cross-sectional study among healthcare professionals
Source: BMC Health Serv Res. 2021 Jun 2;21:541. doi: 10.1186/s12913-021-06430-3 (PMC8171052; doi:10.1186/s12913-021-06430-3)
Supplement: Supplementary file 1 — Additional file 1. IcanSDM – German version. [file 12913_2021_6430_MOESM1_ESM.docx]

**Additional File 3: IcanSDM – German version**

Bitte geben Sie durch ein Kreuz auf der Linie an, wie stark Sie jeder der folgenden Aussagen zustimmen. Geben Sie Ihre persönliche Meinung an und beziehen Sie sich auf Ihren aktuellen Arbeitsplatz.

| 1 | Partizipative Entscheidungsfindung führt zu längeren Gesprächen mit Patientinnen und Patienten.  stimme überhaupt stimme völlig  nicht zu zu |
| --- | --- |
| 2 | Patientinnen und Patienten finden es häufig besser, dass der Arzt/die Ärztin die Entscheidung trifft.  stimme überhaupt stimme völlig  nicht zu zu |
| 3 | Partizipative Entscheidungsfindung kann man weder bei allen Patientinnen und Patienten noch in allen klinischen Situationen anwenden.  stimme überhaupt stimme völlig  nicht zu zu |
| 4 | Es ist zu aufwendig, Patientinnen und Patienten wissenschaftliche Daten zu vermitteln.  stimme überhaupt stimme völlig  nicht zu zu |
| 5 | Partizipative Entscheidungsfindung beansprucht zu viele Ressourcen (z.B. Zeit, Personal).  stimme überhaupt stimme völlig  nicht zu zu |
| 6 | Partizipative Entscheidungsfindung ist nicht mit klinischen Leitlinien vereinbar.  stimme überhaupt stimme völlig  nicht zu zu |
| 7 | Partizipative Entscheidungsfindung ist nur ein vorübergehender Trend.  stimme überhaupt stimme völlig  nicht zu zu |
| 8 | Durch partizipative Entscheidungsfindung erfahren Patientinnen und Patienten, dass Behandlungen mit Unsicherheiten verbunden sind. Dies könnte Patientinnen und Patienten verwirren.  stimme überhaupt stimme völlig  nicht zu zu |
